# Cost savings of reduced constipation rates attributed to increased dietary fiber intakes: a decision-analytic model

**DOI:** 10.1186/1471-2458-14-374

**Published:** 2014-04-17

**Authors:** Jordana K Schmier, Paige E Miller, Jessica A Levine, Vanessa Perez, Kevin C Maki, Tia M Rains, Latha Devareddy, Lisa M Sanders, Dominik D Alexander

**Affiliations:** 1 Exponent Inc., 1800 Diagonal Road Suite 500, Alexandria, VA 22314, USA; 2 Exponent Inc., 525 W. Monroe Street Suite 1050, Chicago, IL 60661, USA; 3 Biofortis–Provident Clinical Research, 211 E. Lake Street #117, Addison, IL 60101, USA; 4 The Kellogg Company, 2 Hamblin Ave, Battle Creek, MI 49015, USA; 5 Exponent Inc., 4141 Arapahoe Avenue Suite 101, Boulder, CO 80303, USA

**Keywords:** Constipation, Public health, Prevention, Cost-savings, Fiber, Diet, Lifestyle

## Abstract

**Background:**

Nearly five percent of Americans suffer from functional constipation, many of whom may benefit from increasing dietary fiber consumption. The annual constipation-related healthcare cost savings associated with increasing intakes may be considerable but have not been examined previously. The objective of the present study was to estimate the economic impact of increased dietary fiber consumption on direct medical costs associated with constipation.

**Methods:**

Literature searches were conducted to identify nationally representative input parameters for the U.S. population, which included prevalence of functional constipation; current dietary fiber intakes; proportion of the population meeting recommended intakes; and the percentage that would be expected to respond, in terms of alleviation of constipation, to a change in dietary fiber consumption. A dose–response analysis of published data was conducted to estimate the percent reduction in constipation prevalence per 1 g/day increase in dietary fiber intake. Annual direct medical costs for constipation were derived from the literature and updated to U.S. $ 2012. Sensitivity analyses explored the impact on adult vs. pediatric populations and the robustness of the model to each input parameter.

**Results:**

The base case direct medical cost-savings was $12.7 billion annually among adults. The base case assumed that 3% of men and 6% of women currently met recommended dietary fiber intakes; each 1 g/day increase in dietary fiber intake would lead to a reduction of 1.9% in constipation prevalence; and all adults would increase their dietary fiber intake to recommended levels (mean increase of 9 g/day). Sensitivity analyses, which explored numerous alternatives, found that even if only 50% of the adult population increased dietary fiber intake by 3 g/day, annual medical costs savings exceeded $2 billion. All plausible scenarios resulted in cost savings of at least $1 billion.

**Conclusions:**

Increasing dietary fiber consumption is associated with considerable cost savings, potentially exceeding $12 billion, which is a conservative estimate given the exclusion of lost productivity costs in the model. The finding that $12.7 billion in direct medical costs of constipation could be averted through simple, realistic changes in dietary practices is promising and highlights the need for strategies to increase dietary fiber intakes.

## Background

Current dietary fiber intakes in the United States are far below recommended levels [1], with recent estimates indicating that less than three percent of men and only six percent of women meet recommendations [2]. Although a universally-accepted definition of dietary fiber does not exist, the Institute of Medicine (IOM) defines dietary fiber as nondigestible carbohydrates and lignin that are intrinsic (i.e., naturally occurring) and intact in plants [1]. To increase dietary fiber intakes, the Dietary Guidelines for Americans, 2010 [3] recommends that Americans should select fiber-rich foods such as whole grains, vegetables, fruits, and beans and peas, while limiting refined grains. In particular, the Dietary Guidelines specify that at least half of all grains consumed should be whole grains. Accumulating evidence suggests that greater dietary fiber intakes reduce risk for type 2 diabetes [4,5], cardiovascular disease [5-7], certain cancers [8-10], weight gain [11,12], diverticular disease [13,14], and obesity [11], as well as functional constipation [15-20].

Reports of the prevalence of functional constipation, also referred to as chronic idiopathic constipation, vary widely, ranging from 1.9 to 27.1% in the general U.S. population [21,22]. These variations result from differences in important factors such as how constipation is defined and how cases are ascertained (e.g., self-report vs. clinical evaluation). Overall, prevalence of functional constipation increases with advancing age and occurs more frequently in women than men [21]. According to the most recent set of Rome diagnostic criteria (Rome III), which are the most widely accepted criteria [23] functional constipation is characterized by defecation associated with straining, hard stools, a sensation of incomplete evacuation, a sensation of anorectal obstruction (requiring manual maneuvers), and less than three stools per week. Functional constipation is commonly considered easily preventable, with increased dietary fiber intake being a safe and effective option. If left untreated, functional constipation may develop into serious bowel disorders with implications for work impairment, lost productivity, reduced health-related quality of life, and rising medical costs [24,25]. For example, from 1958 to 1986, the average number of doctor visits for constipation in the United States was 2.5 million annually [26]. However, the frequency of medical visits for constipation-related care has increased considerably in the last 20 years, thus representing a growing economic burden for both the patient and medical provider, with the number of physician visits totaling 4 million from 1993 through 1996 and 7.95 million from 2001 through 2004 [27].

The objective of this research was to identify potential cost savings, in terms of direct medical expenditures, i.e., medical encounters and prescription and over-the-counter (OTC) laxatives, associated with increased intake of dietary fiber in the United States. To accommodate the multiple sources of uncertainty in the required input parameters, the model was designed to be flexible and capable of exploring a wide range of inputs. A base case as well as multiple sensitivity analyses were tested and are presented herein.

## Methods

The decision-analytic model was developed using and estimates the direct medical cost savings associated with constipation among adults based on the following factors: (1) baseline dietary fiber consumption; (2) prevalence of constipation among those not meeting dietary fiber intake recommendations; (3) proportion of those likely to respond, in terms of alleviation of constipation-associated symptoms, to increased dietary fiber intakes; (4) estimated decrease in constipation prevalence associated with each 1 g/day increase of dietary fiber intake; and (5) expected change in dietary fiber intake. The medical costs prior to and following the hypothetical intervention (i.e., increased dietary fiber intake) are compared. All data included in this manuscript were extracted from the peer-reviewed literature and no original research involving human subjects (including human material or human data) was conducted; therefore, the study was not subject to institutional review board review and did not require written informed consent.

Two age populations are available in the model: children (aged 5 to 17 years of age) and adults (aged 18 years and above). The population of children 4 years of age and younger is excluded from this model because there is limited information on the prevalence of functional constipation among infants and preschoolers [28] and treatment patterns appear to differ for infants and preschoolers compared to older children and adolescents [29]. In addition, the limited available data on treatment costs suggests substantial differences between infants and preschoolers compared to older children, adolescents, and adults (citation) and it is unknown, particularly among infants, if increasing dietary fiber is a potential treatment option. In this model, the user can select *pediatric*, *adult*, or *all* as the population of interest. Children 5 to 17 years are included in the model’s sensitivity analyses, but not in the base case of the model because there is more uncertainty around the model’s input parameters for children, although reasonable estimates were used to populate the model.

### Identification and definition of input parameters

The spreadsheet model includes inputs from multiple sources. A full list of these sources and base case parameters are presented in Table 1. Many of the input parameters were obtained directly from the peer-reviewed literature. As the table demonstrates, default values were provided but the user can vary the input within a specified range or add a value that is more specific or is outside of the range.

**Table 1:** Input parameters for decision-analytic model and corresponding sources

**Parameter**	**Value (Base Case)**	**Source(s)**
Population meeting fiber recommendations		
Adult, male	3%	USDA, 2010 [2]
Adult, female	6%	USDA, 2010 [2]
Pediatric, male	3%	USDA, 2010 [2]
Pediatric, female	3%	USDA, 2010 [2]
Population with constipation		
Adult, male	4.6%	Stewart et al., 1999 [22]
Adult, female	4.6%	Stewart et al., 1999 [22]
Pediatric, male	4.6%	Based on Stewart et al., 1999 [22]
Pediatric, female	4.6%	Based on Stewart et al., 1999 [22]
Percent of population expected to respond to fiber^a^	85%	Voderholzer et al. 1997 [30]
Percent reduction in constipation associated with each 1 g/day increase in fiber intake	1.9%	Dukas et al. 2003 [18]
Constipation severity		
Adults	75% require prescription	Assumption
Pediatric	100% require prescription	Assumption
Annual cost		
Adult, prescription	$10,786.15	Mean of Mitra et al., 2011 [31] and Nyrop et al. 2007 [32], inflated to 2012
Adult, OTC	$566.54	Nyrop et al. 2007 [32], inflated to 2012
Pediatric, prescription	$3032.97	Liem et al., 2009 [33], inflated to 2012
Change in fiber intake	Increase of 9 g daily	Assumption^b^

*Abbreviations*: *USDA* U.S. Department of Agriculture, *OTC* over-the-counter.

^a^Respond to fiber = alleviation of constipation.

^b^Assumed value of 9 g/day corresponds to the difference between the lower limit of the fiber recommendation established by the IOM (25 g/day) and the current mean intake in the population (16 g/day).

Values for baseline dietary fiber intakes were derived from publicly available estimates provided by the U.S. Department of Agriculture (USDA), using data from the National Health and Nutrition Examination Survey (NHANES) 2003–2006 [2]. Intake estimates from NHANES 2003–2006 were used for consistency purposes, as the values for the percentages of the population meeting dietary fiber recommendations, defined by Adequate Intakes (which are intake levels established by the IOM and considered to be adequate for the population) [2], were from NHANES 2003–2006 [2]. Conservative estimates of constipation prevalence, based on Rome diagnostic criteria, for adults and children [22] were identified from a systematic review of the epidemiologic literature available in PubMed through January 2013. Although other estimates exist, they are often based on self-report, which may lead to higher estimates. In an effort to be conservative in quantifying the population on whom fiber intake might affect constipation, this model uses what might be considered a low estimate of constipation prevalence. Only one epidemiologic study identified provided sufficient data to estimate the percentage reduction in constipation prevalence associated with an increase in dietary fiber intake [18]; this study was conducted among women in the Nurses’ Health Study aged 36 to 61 years of age. The dose–response estimate generated from this study was used as the default value in the model (for each 1 gram increase in dietary fiber intake, the prevalence of constipation decreases by 1.9%).

A literature search was conducted to identify the costs of treatment for patients with functional constipation. Several studies were identified [31-35]; they were categorized by the population studied and the treatment required into pediatric, adult OTC, and adult prescription. Given the absence of data for the cost of pediatric non-prescription care, the base case assumes that adult and pediatric OTC annual costs are equivalent. Nevertheless, the default value for pediatric constipation costs assume that treatment costs are derived 100% from prescription and 0% from OTC, as there are limited data on usage of OTC laxatives in children, and there are only anecdotal reports of off-label use of products not approved for children. Only studies from the United States were included, and costs were inflated to 2012 dollars using the Bureau of Labor Statistics’ Medical Care Consumer Price Index [36]. Table 2 presents sources for cost inputs for the model.

**Table 2:** Results and sensitivity analyses: cost savings of reduced constipation rates attributed to increased dietary fiber intakes

**Annual cost reduction**	**Key parameters and/or change(s) in key parameters**
$12.7 billion	Base case
$1.1 billion	No change in fiber intake for 75% of adults; 25% of adults increase fiber intake by 3 g daily
$13.2 billion	Assumes that 1% of men and women currently meet fiber intake recommendation
$12.0 billion	Assumes that 10% of men and women currently meet fiber intake recommendation
$7.5 billion	Assumes that 50% of adults with constipation respond to fiber increase
$4.8 billion	Assumes that 25% of adults with constipation require a prescription; 75% take over-the counter products
$20.0 billion	Assumes that 1 g of increased fiber intake is associated with a 3% reduction in constipation
$19.3 billion	Assumes that 7% of the adult population has constipation
$2.8 billion	Assumes that 1% of the adult population has constipation
$19.5 billion	Assumes that 4.0% of men and 10.2% of women have constipation {Markland, 2013 #632}
$21.9 billion	Assumes that adults increase fiber intake by 15 g daily
$0.7 billion	Pediatric population only; assumes 100% of the population increased fiber intake by 6 g daily
$83.9 billion	Multivariate: best case - Assumes that 1% of adults meet fiber intake recommendations, 7% of adults have constipation, 1 g of fiber intake is associated with a 3% reduction in constipation, all patients require a prescription medication, 100% of adults increase fiber intake (by 15 g daily)
$2.3 million	Multivariate: worst case - Assumes that 10% of adults meet fiber intake recommendations, 1% of adults have constipation, 1 g of fiber intake is associated with a 1% reduction in constipation, all patients are treated with an over the counter medication, only 25% of adults increase fiber intake (by 3 g daily)

A base case was created to reflect a plausible scenario, given existing information. The base case is limited to the adult population; for simplicity, assumptions for adult and pediatric populations are presented here. For each input parameter, sensitivity analyses were conducted, assuming a range of plausible values. In the case of costs, the base case values were increased and decreased by 50%. One highly variable parameter required for the model is what percent of the population will make particular dietary changes. The model is designed to allow the user to specify the distribution of the population that falls into the following categories: no additional dietary fiber, or an additional 3, 5, 6, 7.5, 8, 9, 10, or 15 g/day of dietary fiber. The base case assumed that all adults with dietary fiber intake below recommended levels would increase their dietary fiber intakes by 9 g/day, a value which corresponds to the difference between the lower limit of the dietary fiber recommendation established by the IOM (25 g/day) [1] and the current mean intake (16 g/day) [2]. The lower limit of the dietary fiber recommendation for adult women was selected as a conservative estimate, as the lower end of the recommended dietary fiber intake range for adult men is 38 g/day [1]. The value of 25 g/day also aligns with the Daily Value used on the Nutrition Facts Panel for a 2000 calorie diet. The proportion of cases treated with prescription or OTC medications is an assumption, based on review of published case series. The base case assumes that 75% of adults who present for care require a prescription and that 100% of children require one. These values can be modified by the user and were tested in sensitivity analyses.

## Results

Table 1 lists the input parameters used for the base case of the model as well as the corresponding parameters for the pediatric population. For example, the base case assumes that 3% of the adult male population and 6% of the adult female population meet dietary fiber recommendations [2] and that 4.6% of adults (male and female) have functional constipation [22]. Sources are provided for each parameter.

Table 2 presents results for the base case of the model and selected sensitivity analyses. Under the base case assumptions, the annual cost savings were estimated to be $12.7 billion. Sensitivity analyses were based on varying the input parameters. The table presents the annual cost reduction associated with each different scenario. As expected, results from the sensitivity analyses showed variation across the different input parameters, but all plausible scenarios resulted in cost savings of at least $1 billion, with maximum savings associated with a successful public health campaign that would result in substantially increased dietary fiber intakes. With this assumption of adults increasing dietary fibers intakes by 15 g/day, more than $80 billion in annual savings could be achieved. The proportion of adults with constipation in the base case was set to 4.6% for all adults; a recent U.S. study using a different methodology for identifying constipation rates has suggested this value may be too low for women. Thus, a sensitivity analysis used values from the most recent NHANES analysis, which estimated rates were 4.0% for men and 10.2% for women [37]. No input parameter was identified as trivial, nor was any parameter identified that dominated the sensitivity analysis. As shown in Table 2, if only 1% of adults currently meet their dietary fiber intake recommendation and all remaining individuals who are not meeting recommendations increase their intake by 9 g daily, then the savings increases slightly to $13.2 billion from the base case of $12.7 billion. If 10% of adults already meet the recommendation, the savings, as expected, is slightly smaller, at $12.0 billion. The last two rows in Table 2 present results of multivariate sensitivity analyses of best and worst case scenarios. The best case scenario has the lowest proportion of the population meeting dietary fiber intake recommendations, the highest rates of constipation, and the best response to dietary fiber intake; this scenario results in estimated savings of more than $83 billion annually. The worst case scenario assumes the opposite, that is, the highest rates of dietary fiber intake along with the lowest rates of constipation and the lowest constipation costs (i.e. use of over-the-counter medications only). This scenario results in a modest cost savings of just greater than $2 million. An analysis of the potential cost savings among the pediatric population projected a savings of $0.7 billion annually.

## Discussion

Increased dietary fiber consumption for adults with functional constipation is associated with a considerable cost savings, likely exceeding $12 billion, with a possibility of exceeding $20 billion with small changes in the model. The inclusion of the pediatric population adds another small but substantial increment ($0.7 billion). The finding that tens of billions of dollars in constipation costs could be averted through simple, realistic changes in dietary practices is promising and highlights the need for strategies to increase dietary fiber intakes. These strategies should include efforts to ensure all individuals have access to fiber-rich foods as well as efforts to modify individual dietary intake behavior (e.g., providing nutrition education and increasing awareness of the health benefits of dietary fiber).

An important consideration from an economic standpoint relates to the types of foods that individuals would consume to obtain additional dietary fiber in their diets. This analysis did not assume an additional cost for increased dietary fiber consumption. Many foods that are available in both conventional and fiber-enriched versions, such as ready-to-eat cereal and snack bars, are comparably priced; in addition, there is a lack of data showing that fiber-rich foods are more expensive than lower-fiber foods [14]. Therefore, an additional cost was considered unnecessary for the model. In other words, the model assumes that consumers make simple substitutions of one product for another similarly-priced product that is otherwise nutritionally similar. This is supported by findings from a recent study that demonstrated how fiber intakes can be increased with simple, small-step substitutions without affecting caloric intake [38]. However, it is possible that a different pattern of consumption would occur, i.e., consumers may replace calorie-dense food items with low energy-dense food items. For example, consumers may replace energy-dense candy bars with fiber-rich cereal bars. In this case, dietary fiber intake is not the only factor that changes, but intakes of total energy and saturated fat may decrease, while intakes of other dietary components generally found in fiber-rich foods, such as magnesium [39], may increase. Furthermore, overall diet quality may improve as a result of replacing calorie-dense foods items with lower energy-dense, fiber-rich food items [13,40]. The implications are that there can be many other related health and economic outcomes affected. A substitution of a similar product might yield results such as this model calculated; in contrast, the replacement of one type of food for a dissimilar one may mean this model, focused only on constipation, grossly underestimates savings in direct medical costs that result from the unintended consequence of eliminating other products from the diet in favor of fiber. A related consideration is that not all types of dietary fiber are equally effective at enhancing regularity. For example, wheat bran fiber in particular has been shown to promote bowel regularity [39,41]. However, the assumptions for the model were based on data from the Nurses’ Health Study [18], which evaluated total dietary fiber. Should other data sources or estimates become available, these can be incorporated as new input parameters into the existing model. In addition, the current model allows for the exploration of a number of possibilities, including varying dietary fiber intakes and reductions in constipation prevalence associated with each additional gram of dietary fiber intake.

Some considerations are warranted for the basis of the model parameters used in this study. Regarding the epidemiologic data used for input parameters, misclassification, misdiagnosis, and failure to diagnose (as not all individuals who suffer from constipation seek medical care) have hampered efforts to ascertain accurate prevalence and incidence of functional constipation. Even when validated surveys have been used to define constipation, the estimates can differ greatly based on method and whether frequency, consistency, or a combination of the two are considered [42]. By using a fairly strict criterion for constipation, these findings may underestimate the number of potential patients and thus the potential cost savings. In addition, certain populations are often excluded from the types of surveys that estimate prevalence, including the institutionalized elderly and neonates, as well as patients who are suffering from opioid- or other drug-induced constipation or for patients where polypharmacy is necessary [43-45]. The diagnostic criteria for constipation have also changed over time, and dietary fiber intake estimates are based on self-report. Furthermore, the estimate used for the decrease in constipation associated with each 1 g/day increase in dietary fiber intake was derived from a single study of adult women; further analysis to refine the value or to provide separate estimates by age and sex would be welcomed. Finally, population-level assumptions were made about baseline dietary fiber intake and constipation prevalence. No data were available to identify dietary fiber intake for those with and without constipation.

Regarding the economic input parameters and calculations, it should be noted that the model assumes that a 1.9% decrease in constipation would result in a 1.9% reduction in constipation-related costs. Although plausible, this assumption does not account for a variety of scenarios. For instance, this change in constipation might decrease the severity of constipation from requiring a prescription to being treated by OTCs, or it might move an individual who uses OTCs often to using them rarely, or it might mean those with already close-to-acceptable dietary fiber intake might have no constipation costs at all. More likely it means a combination of these possibilities will exist in the population rather than just one. Last, cost estimates are sparse and limited to direct medical costs, without considering important indirect costs such as those related to lost work or school days per month [46,47]. Therefore, limiting the model to direct medical costs savings yields a conservative estimate for constipation-related cost. Nonetheless, the model developed here demonstrates a considerable cost savings associated with a simple, feasible, and realistic approach to reduce the economic burden from constipation, namely increasing dietary fiber consumption.

## Conclusion

The public health implications of increasing dietary fiber intake to recommended levels for gastrointestinal health and chronic disease prevention are significant. Accumulating evidence indicates that greater dietary fiber intakes reduce risk for type 2 diabetes [4,5], cardiovascular disease [5-7], certain cancers [8-10], weight gain [11,12], obesity [11], and diverticular disease [13,14], as well as functional constipation [15-20]. In 2010, the Dietary Guidelines for Americans classified dietary fiber as a nutrient of concern because more than 90% of U.S. adults and children failed to meet their daily dietary fiber recommendations [3]. Given that the vast majority of Americans are not meeting recommendations yet the potential benefits of increasing intakes for both public health and cost savings are substantial, more comprehensive efforts are needed to align intakes with recommendations. These efforts should include strategies to increase the supply, availability, and consumer demand of fiber-rich foods, such as whole grains, vegetables, nuts, and legumes, and to provide higher fiber varieties of the foods individuals are presently consuming, including ready-to-eat cereals.

## Abbreviations

IOM: Institute of medicine; NHANES: The national health and nutrition examination survey; OTC: Over-the-counter; USDA: United States Department of Agriculture.

## Competing interests

JKS, PEM, JAL, VP, and DDA are employed at Exponent, Inc. and KCM and TMR are employed at Biofortis-Provident Clinical Research. Funding from Kellogg Company directly supported the efforts of KCM and TMR, and Exponent received funding to conduct this research through Biofortis-Provident Clinical Research.

## Authors’ contributions

JKS, DDA, KCM, LMS and TMR conceptualized the study; JKS and PEM designed the study plan; and JKS and JAL developed the economic model, with support from PEM and VP in obtaining input parameters. All authors interpreted and discussed the study findings. JKS, PEM, and JAL wrote the manuscript, with critical revision and feedback for intellectual content from VP, KCM, TMR, LD, LMS, and DDA. All authors read and approved the final version of this manuscript.

## Authors’ information

At the time this research was completed, Dr. Miller and Dr. Alexander were employed at Exponent.

## Pre-publication history

The pre-publication history for this paper can be accessed here:

http://www.biomedcentral.com/1471-2458/14/374/prepub
